# Renin-angiotensin-aldosterone system blockade is associated with higher risk of contrast-induced acute kidney injury in patients with diabetes

**DOI:** 10.18632/aging.102982

**Published:** 2020-04-02

**Authors:** Mengqing Ma, Xin Wan, Min Gao, Binbin Pan, Dawei Chen, Qing Sun, Mengyu Zhang, Changgao Zhou, Tao Li, Hanchao Pan, Wei Shao, Zhihe Liu, Yue Chen, Changchun Cao

**Affiliations:** 1 Department of Nephrology, Sir Run Run Hospital, Nanjing Medical University, Nanjing 211166, Jiangsu, China; 2 Department of Nephrology, Nanjing First Hospital, Nanjing Medical University, Nanjing 210006, Jiangsu, China; 3 Department of Nephrology, Xu Zhou Medical University Hospital, Xuzhou 221000, Jiangsu, China; 4 Department of Cardiology, Affiliated Shu Yang Hospital, Nanjing University of Traditional Chinese Medicine, Shuyang 223600, Jiangsu, China

**Keywords:** contrast-induced acute kidney injury, diabetes mellitus, angiotensin-converting enzyme inhibitors, angiotensin receptor blockers, coronary angiography

## Abstract

As the incidence of diabetes and cardiovascular comorbidities continues to rise, driven by increased prevalence of obesity and an aging population, so does the demand for percutaneous coronary intervention (PCI) to restore cardiac blood flow. Renin-angiotensin-aldosterone system (RAAS) inhibitors are commonly prescribed to hypertensive diabetic patients to prevent diabetic nephropathy. However, evidence suggests that angiotensin-converting enzyme inhibitors (ACEIs) and angiotensin receptor blockers (ARBs) may increase the risk of contrast-induced acute kidney injury (CIAKI) following coronary angiography (CAG) and PCI. We therefore conducted a retrospective, multicenter study applying the propensity score matching method to evaluate the impact of RAAS inhibition on CIAKI in diabetic patients undergoing CAG/PCI. Among 2240 subjects that met the inclusion criteria, 704 patients in the ACEIs/ARBs group were successfully matched to eligible control patients. The incidence of CIAKI (serum creatinine increase ≥0.5 mg/dl or ≥25% from baseline within 72 h post-CAG/PCI) was significantly higher in the ACEIs/ARBs group than in the control group (26.6% vs. 16.2%, *P*<0.001). However, control patients showed increased risk of overall adverse cardiovascular events (4.1% vs. 1.8% for ACEIs/ARBs; *P*=0.016). These data indicate that RAAS inhibition increases the risk of CIAKI in diabetic patients, but confers protection against early cardiovascular events.

## INTRODUCTION

The incidence of diabetes and coronary heart disease continues to rise as a result of the current obesity epidemics and an increasingly aging population worldwide. Consequently, we are seeing a steady increase in the number of percutaneous coronary intervention (PCI) procedures performed to restore blood supply to the heart [1, 2]. Contrast-induced acute kidney injury (CIAKI) is a common complication after PCI, and is associated with significant short- and long-term morbidity and mortality [3]. The incidence of CIAKI varies widely depending on the different definition criteria, study populations, and prevention strategies [4]. In the general population, CIAKI occurs in less than 3% of patients undergoing PCI, whereas in higher-risk populations such as those with diabetes or renal failure, the incidence can be as high as 50% [5]. Diabetes is one of the most important and common risk factors for CIAKI [6]. In diabetic patients complications develop 3 times faster than in those without the disease [7], and CIAKI-related mortality rates can be as high as 30% [8].

Renin-angiotensin-aldosterone system (RAAS) inhibitors [i.e. angiotensin converting enzyme inhibitors (ACEIs) and angiotensin receptor blockers (ARBs)] are commonly used to reduce blood pressure and preserve renal function [9, 10]. RAAS inhibitors are also commonly prescribed to hypertensive patients with diabetes or chronic kidney disease (CKD) to reduce urinary albumin excretion and prevent or delay the onset of diabetic nephropathy and end-stage renal disease (ESRD) [11–13]. However, despite the proven benefits of long-term administration of ACEIs/ARBs in these settings, clinicians have become aware of several potential unwanted effects. For instance, the latest ACC/AHA High Blood Pressure Clinical Practice Guideline has pointed out that in patients with hypertension undergoing major surgery, especially cardiac surgery, preoperative discontinuation of ACEIs/ARBs may be considered [11]. The relationship between ACEIs/ARBs and CIAKI remains controversial, since several studies indicated that RAAS blockers are independent risk factors for the occurrence of CIAKI [14, 15], while other investigations showed opposite results [16, 17]. Thus, guidelines issued by the Contrast Media Safety Committee of the European Society of Urogenital Radiology (ESUR) have indicated that there is insufficient evidence to determine whether ACEIs should be discontinued or not before surgery, and stressed the need for further research [18]. Therefore, we conducted a retrospective multicenter research study that assessed the impact of RAAS blockers on CIAKI incidence in diabetic patients undergoing coronary angiography (CAG) and PCI.

## RESULTS

### Baseline clinical characteristics

A total of 2,240 diabetic patients (1,525 from Nanjing First Hospital and 715 from 3 other hospitals) who underwent PCI treatment met the inclusion criteria. The basic characteristics of all patients before and after PSM are listed in Table 1. Before matching, patients in the ACEIs/ARBs group had relatively higher preoperative systolic blood pressure. The prevalence of hypertension, proteinuria, unstable angina, multi-vessel disease, and use of β-blockers, oral hypoglycemic agents, and diuretics was also higher in patients receiving ACEIs/ARBs compared with controls. By propensity score, 704 patients in the ACEIs/ARBs group were successfully matched to an equal number of eligible patients in the control group. There were no significant differences in baseline characteristics between the two groups. As for the unmatched patients, those in the ACEIs/ARBs group had higher preoperative systolic and diastolic blood pressure, and lower estimated glomerular filtration rate (eGFR) than controls. In turn, prevalence of hypertension, CKD, acute myocardial infarction (AMI), prior myocardial infarction, unstable angina, multi-vessel disease, β-blockers, diuretics, calcium channel blockers (CCB), oral hypoglycemic agents, and proteinuria were also higher in the ACEI/ARB group. The characteristics of the patients from the 4 participating medical centers are listed in Supplementary Tables 1 and 2. The characteristics of patients before and after merging each matched center are listed in Supplementary Table 3.

**Table 1:** Baseline characteristics of patients in all centers.

**Variable**	**Before matching**			**After propensity matching**			**The rest after matching**		
	**ACEI/ARB group (n=1310)**	**Control group (n=930)**	***P* value**	**ACEI/ARB group (n=704)**	**Control group (n=704)**	***P* value**	**ACEI/ARB group (n=606)**	**Control group (n=226)**	***P* value**
**Demographics:**									
Female	458(35.0)	311(33.4)	0.455	239(33.9)	231(32.8)	0.685	219(36.1)	80(35.4)	0.843
Age (yrs)	66±10	66±11	0.238	66±10	66±10	0.777	66±10	63±11	0.312
BMI (kg/m^2^)	25.4±3.1	24.9±3.0	0.381	25.2±3.0	25.1±3.0	0.595	25.6±3.1	24.3±2.8	0.367
**Medical history:**									
Diabetes history (yrs)	8.2±5.8	8.3±6.0	0.433	8.6±5.9	8.4±6.2	0.584	7.8±5.8	7.8±5.8	0.845
Hypertension	1146(87.5)	547(58.8)	**<0. 001**	550(78.1)	537(76.3)	0.255	596(98.3)	10(4.4)	**<0. 001**
CHF	195(14.9)	132(14.2)	0.648	94(13.4)	103(14.6)	0.538	101(16.7)	29(12.8)	0.175
CKD	181(13.8)	108(11.6)	0.125	95(13.5)	92(13.1)	0.877	86(14.2)	16(7.1)	**0.005**
AMI	274(20.9)	222(23.9)	0.097	141(20.0)	156(22.2)	0.361	133(21.9)	66(29.2)	**0.029**
Prior myocardial infarction	106(8.1)	64(6.9)	0.287	51(7.2)	57(8.1)	0.624	55(9.1)	7(3.1)	**0.003**
Stable angina pectoris	81(6.2)	66(7.1)	0.390	54(7.7)	52(7.4)	0.919	27(4.5)	14(6.2)	0.303
Unstable angina	525(40.1)	323(34.7)	**0.010**	278(39.5)	260(36.9)	0.340	247(40.8)	63(27.9)	**0.001**
**CAG and PCI:**									
Multi-vessel disease	797(60.8)	512(55.1)	**0.006**	401(57.0)	399(56.7)	0.957	396(65.3)	113(50.0)	**<0. 001**
Single-vessel disease	390(29.8)	293(31.5)	0.380	219(31.1)	220(31.3)	1.000	171(28.2)	73(32.3)	0.250
Preoperative SBP (mmHg)	137±17	131±17	**0.017**	134±16	134±17	0.828	142±18	121±14	**<0. 001**
Preoperative DBP (mmHg)	80±12	78±11	0.685	78±10	79±11	0.567	83±13	74±10	**0.006**
**Contrast agent:**									
Nonionic iso-osmolar	638(48.7)	444(47.7)	0.654	350(49.7)	348(49.4)	0.959	288(47.5)	96(42.5)	0.194
Nonionic low-osmolar	657(50.2)	479(51.5)	0.528	347(49.3)	349(49.6)	0.959	310(51.2)	130(57.5)	0.102
Volume of contrast agent (mL)	184±76	179±74	0.681	183±74	183±77	0.867	185±78	166±61	0.405
**Medications :**									
Β-blocker	843(64.4)	439(47.2)	**<0. 001**	382(54.3)	360(51.1)	0.193	461(76.1)	79(35.0)	**<0. 001**
Diuretics	330(25.2)	143(15.4)	**<0. 001**	122(17.3)	124(17.6)	0.942	208(34.3)	19(8.4)	**<0. 001**
CCB	326(24.9)	213(22.9)	0.280	204(29.0)	201(28.6)	0.904	122(20.1)	12(5.3)	**<0. 001**
Insulins	584(44.6)	419(45.1)	0.824	327(46.4)	332(47.2)	0.827	257(42.4)	87(38.5)	0.308
Oral hypoglycemic agent	764(58.3)	496(53.3)	**0.019**	385(54.7)	385(54.7)	1.000	379(62.5)	111(49.1)	**<0. 001**
**Pre-procedural laboratory determinations:**									
Glucose (mmol/L)	9.6±3.6	9.6±3.9	0.183	9.6±3.5	9.5±3.6	0.541	9.5±3.7	9.9±4.3	0.124
Baseline creatinine (umol/L)	77.3±29.2	76.5±34.2	0.750	77.6±31.6	77.7±33.0	0.969	76.9±26.1	72.7±37.6	0.819
eGFR (mL/min/1.73 m^2^)	84.4±20.8	86.3±20.9	0.419	84.6±20.9	84.8±20.9	0.912	84.1±20.6	91.1±20.1	**0.045**
Proteinuria	207(15.8)	105(11.3)	0.002	80(11.4)	84(11.9)	0.804	127(21.0)	21(9.3)	**<0. 001**
Hemoglobin (g/L)	132.1±16.7	132.6±16.8	0.831	132±17	132±17	0.594	132±17	134±17	0.975
Albumin (g/L)	39.3±4.0	38.9±4.4	0.260	39.1±3.9	39.0±4.5	0.566	39.6±4.1	38.7±3.8	0.165
Uric acid (umol/L)	338.7±110.6	328.1±109.9	0.273	332.0±110.4	335.0±107.1	0.611	346.6±110.4	307±116.0	0.174
Total cholesterol (mmol/L)	4.0±1.2	4.0±1.2	0.899	3.9±1.1	3.9±1.1	0.763	4.1±1.2	4.1±1.3	0.543
Triglycerides (mmol/L)	1.9±1.5	1.8±1.4	0.318	1.8±1.6	1.8±1.3	0.585	1.9±1.5	1.9±1.7	0.576
HDL (mmol/L)	1.01±0.26	1.02±0.26	0.783	1.01±0.25	1.01±0.26	0.971	1.01±0.26	1.02±0.27	0.574
LDL (mmol/L)	2.33±0.92	2.34±0.94	0.756	2.30±0.87	2.31±0.89	0.697	2.38±0.98	2.44±1.06	0.796
LVEF (%)	58.4±9.8	58.6±9.7	0.495	59.0±9.5	58.6±9.7	0.438	57.9±10.1	58.5±9.6	0.323

Abbreviations: ACEI, angiotensin-converting enzyme inhibitor; ARB, angiotensin receptor blocker; BMI, body mass index; CKD, chronic kidney disease; CHF, congestive heart failure; AMI, acute myocardial infarction; CCB, calcium channel blocker; eGFR, estimated glomerular filtration rate; HDL, high-density lipoprotein; LDL, low-density lipoprotein; LVEF, left ventricular ejection fraction.

### RAAS blocker therapy is an independent risk factor for CIAKI

**Figure 1:**
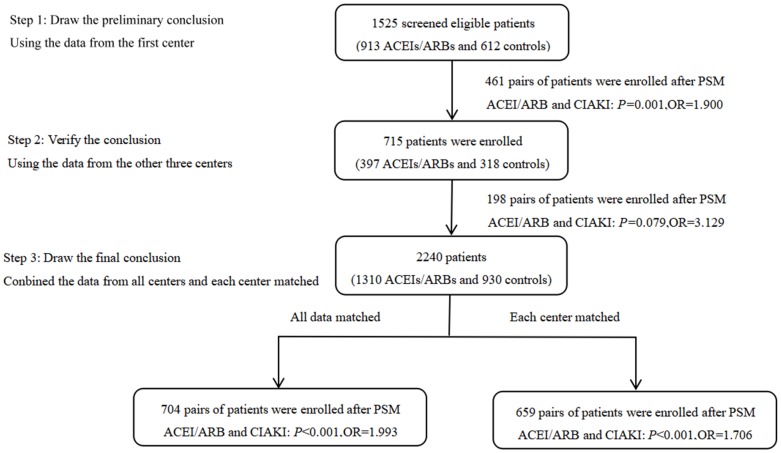
**Summary of study design, methods, and results.** Propensity score matching (PSM) was conducted on 1310 ACEIs/ARBs patients and 930 controls from four medical centers, resulting in 704 patient pairs. After merging matched data from each center, 659 patient pairs were obtained. The conditional logistic model was used to evaluate the association between ACEIs/ARBs use and CIAKI incidence.

**Figure 2:**
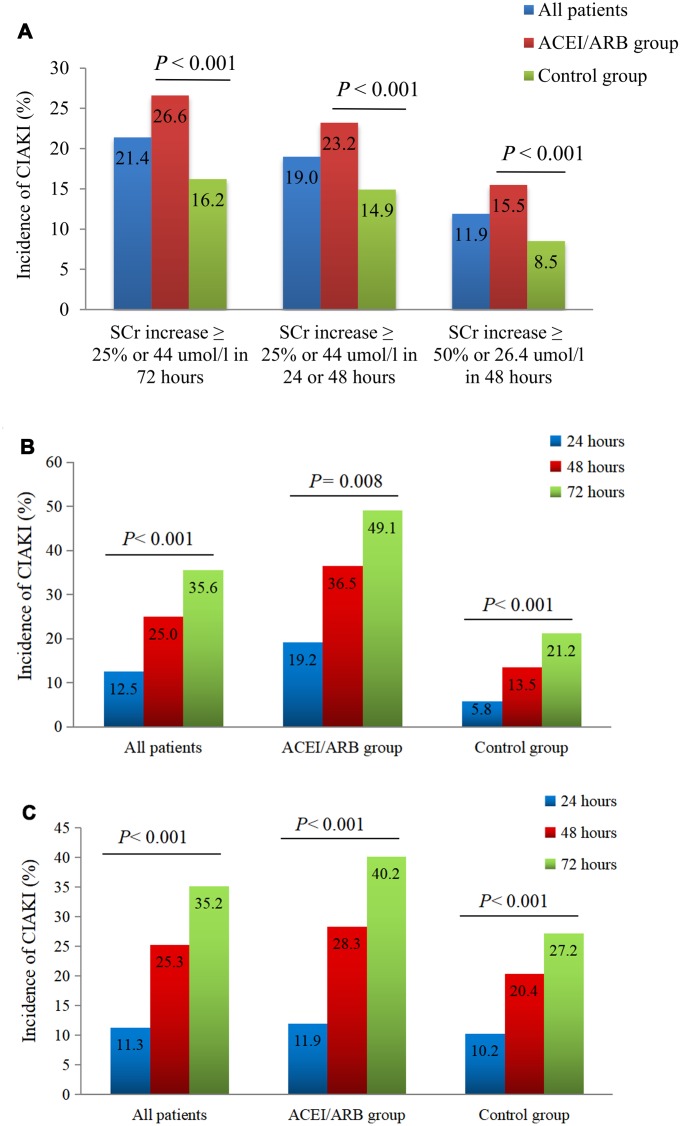
**Impact of RAAS inhibition on CIAKI incidence.** (**A**) Incidence of CIAKI in the PSM-matched cohort under different definitions. (**B**) Incidence of CIAKI in the matched cohort at different times post-CAG/PCI. We screened 52/704 pairs of patients within the PSM-matched cohort who had serum creatinine values documented at 24, 48, and 72 h post-procedure. (**C**) Incidence of CIAKI in the unmatched cohort at different times post-CAG/PCI. We screened 613/2240 patients who had serum creatinine values documented at 24, 48, and 72 h post-procedure.

**Figure 3:**
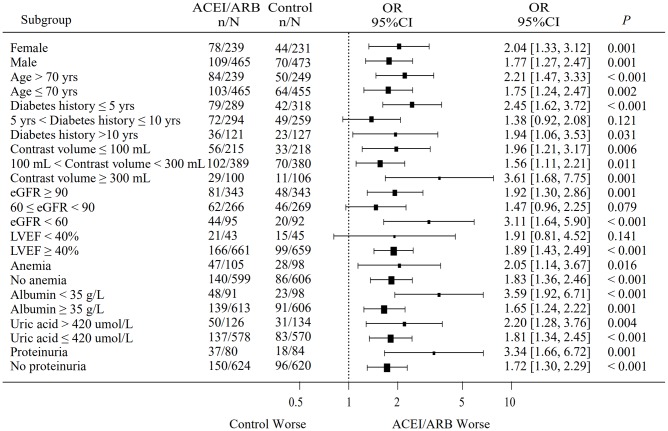
**Subgroup analysis of the effect of RAAS blockers on CIAKI incidence in the matched cohort.** n = number of patients with CIAKI; N = total number of patients in each subgroup; eGFR, estimated glomerular filtration rate; LVEF, left ventricular ejection fraction.

Conditional logistic regression analysis performed in the total matched patient sample indicated that ACEI/ARB use was a risk factor for CIAKI (OR: 1.993, 95% CI: 1.415-2.809; *P*<0.001), and remained a risk factor after PSM-matched data for 659 pairs of patients were merged (OR: 1.706, 95% CI: 1.295-2.246; *P*<0.001) (Figure 1). The incidence of CIAKI, no matter which definition was used, was significantly higher in the ACEIs/ARBs group than in the control group (26.6% vs. 16.2%, *P*<0.001) (Figure 2A; Supplementary Figure 1). Under the different definitions, the two groups in the matched cohort were analyzed based on the conditional logistic regression. Results revealed that RAAS blockade was an independent risk factor for CIAKI (OR: 1.993, 95% CI: 1.415-2.809; *P*<0.001). Meanwhile, multivariate logistic regression analysis showed that ACEIs/ARBs increased the likelihood of developing CIAKI in the unmatched cohort (OR: 1.757, 95% CI: 1.401-2.203; *P*<0.001) (Table 2 and Supplementary Table 4). Additional independent risk factors for CIAKI included female gender, age >70 years, congestive heart failure (CHF), AMI, diabetes history, multi-vessel disease, eGFR, CKD, contrast agent dose, anemia, proteinuria, albumin <35 g/l, uric acid >420 μmol/l, and left ventricular ejection fraction (LVEF) <40% (Table 3). Use of ACEIs/ARBs also increased the incidence of CIAKI in various patient subgroups, especially in those with high risk factors such as age >70 yrs (OR: 2.21, 95% CI: 1.47-3.33; *P*<0.001), contrast volume ≥300 mL (OR: 3.61, 95% CI: 1.68-7.75; *P*=0.001), eGFR <60 mL/min/1.73 m^2^ (OR: 3.11, 95% CI: 1.64-5.90; *P*<0.001), anemia (OR: 2.05, 95% CI: 1.14-3.67; *P*=0.016), albumin <35 g/L (OR: 3.59, 95% CI: 1.92-6.71; *P*<0.001), uric acid > 420 μmol/l (OR: 2.20, 95% CI: 1.28-3.76; *P*=0.004) and proteinuria (OR: 3.34, 95% CI: 1.66-6.72; *P*=0.001) (Figure 3).

**Table 2:** The relationship between ACEI/ARB and CIAKI before and after matching all patients.

**Definitions**	**Unmatched cohort**		**Matched cohort**	
	OR (95% CI)^*^	*P* value^*^	OR (95% CI)^**^	*P* value^**^
**Primary CIAKI end point:**				
SCr increase ≥ 25% or 44 umol/l in 72 hours	1.757 (1.401-2.203)	<0.001	1.993 (1.415-2.809)	<0.001
**Other defining criteria for CIAKI:**				
SCr increase ≥ 25% or 44 umol/l in 24 or 48 hours	1.583 (1.259-1.990)	<0.001	1.725 (1.209-2.460)	<0.001
SCr increase ≥ 50% or 26.4 umol/l in 48 hours	2.009 (1.510-2.673)	<0.001	2.695 (1.672-4.343)	<0.001

^*^Multivariable analysis was applied in the unmatched cohort. OR and 95% confidence interval (CI) were obtained by adjusting variables.

^**^Conditional logistic model was applied in the matched cohort, OR with 95% confidence interval (CI) was obtained.

Abbreviations: SCr, serum creatinine; CIAKI, contrast-induced acute kidney injury.

**Table 3:** Multivariable analysis determining the predictors of primary outcome CIAKI in the unmatched cohort.

**Variable**	**OR**	**95% CI**	***P* value**
Female	1.540	1.229-1.929	<0.001
Age > 70 yrs	1.555	1.212-1.995	0.001
CHF	1.787	1.334-2.394	<0.001
AMI	1.937	1.508-2.489	<0.001
Diabetes history	1.023	1.006-1.042	0.010
Multi-vessel disease	1.216	0.967-1.528	0.094
ACEI/ARB	1.757	1.401-2.203	<0.001
Contrast agent does	0.999	0.997-1.000	0.079
eGFR	1.019	1.009-1.028	<0.001
CKD	2.074	1.310-3.284	0.002
Anemia	1.944	1.443-2.620	<0.001
Albumin < 35 g/L	1.600	1.179-2.170	0.003
Uric acid > 420 umol/L	1.673	1.265-2.213	<0.001
Proteinuria	1.389	1.037-1.860	0.027
LVEF < 40%	1.480	0.991-2.212	0.056

Abbreviations: ACEI, angiotensin-converting enzyme inhibitor; ARB, angiotensin receptor blocker; BMI, body mass index; CKD, chronic kidney disease; CHF, congestive heart failure; AMI, acute myocardial infarction; CCB, calcium channel blocker; eGFR, estimated glomerular filtration rate; HDL, high-density lipoprotein; LDL, low-density lipoprotein; LVEF, left ventricular ejection fraction.

### Impact of ACEIs/ARBs on CIAKI onset and other outcomes

The total incidence of CIAKI after PSM adjustment was 21.4%, which was higher than for other definitions (19.0% and 11.9%) (Figure 2A). There were statistical differences in the incidence of CIAKI (defined as an increase in serum creatinine ≥44 μmol/l (0.5 mg/dl) or 25% or more from baseline) at different time points (24, 48, and 72 h) post-procedure. Thus, CIAKI manifested at higher rates within 24-48 h, compared to the 48-72 h post-CAG/PCI interval (Figure 2B and 2C).

Death occurred in 3 patients from the control group and in 1 patient from the ACEIs/ARBs group (*P*=0.625). One patient in each group needed dialysis (*P*=1.000). Stroke occurred in 3 patients in the control group and in 2 patients in the ACEIs/ARBs group (*P*=1.000). Myocardial infarction occurred in 6/301 patients (2.0%) with CIAKI and in 12/1107 patients (1.1%) without CIAKI (*P*=0.213). Worsening heart failure was documented in 7/301 patients (2.3%) with CIAKI and in 11/1107 patients (1.0%) without CIAKI (*P*=0.068), and RAAS blockade had no impact on these variables. In addition, our study showed no differences for patients treated with or without RAAS blockers in length of in-hospital stay. However, patients in the control group had a higher risk of overall adverse cardiovascular events (death, myocardial infarction -new-onset or recurrence-, worsening heart failure, and stroke) after PCI (4.1% vs. 1.8%, *P*=0.016) (Table 4; Supplementary Table 5).

**Table 4:** Comparison of in-hospital outcomes between the control group and the ACEI/ARB group in the matched cohort (704 pairs of patients).

**Outcome**	**Control group (n=704)**	**ACEI/ARB group (n=704)**	***P* value**
CIAKI, n (%)	114 (16.2)	187 (26.6)	< 0.001
Dialysis, n (%)	1 (0.1)	1 (0.1)	1.000
Deaths, n (%)	3 (0.4)	1 (0.1)	0.625
Worsening heart failure, n (%)	10 (1.4)	5 (0.7)	0.302
Myocardial infarction, n (%)	13 (1.8)	5 (0.7)	0.096
Stroke, n (%)	3 (0.4)	2 (0.3)	1.000
Overall adverse cardiovascular events (at least 1)	29(4.1)	13(1.8)	0.016
Length of in-hospital stay, d	7.94±4.1	8.23±4.1	0.169

Abbreviations: CIAKI, contrast-induced acute kidney injury

## DISCUSSION

Our multi-center study, conducted on a total of 2240 diabetic patients, indicated that RAAS blocker therapy was an independently risk factor for CIAKI, both before and after matching RAAS-blocker users and non-users via PSM analysis (n=704 patient pairs or n=659 patient pairs). Some researchers also found that patients receiving ACEIs/ARBs developed CIAKI more often than those who did not take these medications [19, 20]. The Dialysis-versus-Diuresis (DVD) trial showed that the incidence of CIAKI in a general hospital population was significantly higher in patients treated with RAAS inhibitors (11.9 vs. 4.2%, *P*=0.006) [21]. Results from a prospective cohort study that evaluated the effect of RAAS blockers on CIAKI in diabetic patients with renal dysfunction showed that RAAS treatment was an independently risk factor for CIAKI with an OR value of 2.7 [22]. In addition, in a similar prospective study, Cirit et al. reported that RAAS blockade significantly increased CIAKI incidence in 230 patients with CKD [23]. Meanwhile, inhibition of renin expression with a vitamin D3 analog counteracted the increase in contrast-induced nephropathy induced by the ARB losartan in a rat model of CIAKI [24]. Nevertheless, the influence of RAAS blockers on CIAKI incidence is still controversial. For instance, in a small sample study, Gupta et al. found that the ACEI captopril was a protective factor for CIAKI in 71 patients with diabetes [25]. Meanwhile, a recent study involving 1254 patients with CKD indicated that RAAS blockade reduced the occurrence of CIAKI when a moderate periprocedural hydration volume-to-weight ratio (10.21 to <17.86 mL/kg) was implemented [26]. On the other hand, Spatz et al. reported a retrospective study of 178 patients with CKD that concluded that RAAS blockade had no significant association with CIAKI, and this was also true for diabetic participants [27]. Meanwhile, a randomized prospective trial reported by Rosenstock et al. and conducted on 283 patients with stage 3-4 CKD concluded that withdrawing ACEIs or ARBs 24 h prior to CAG did not modify CIAKI incidence [28]. However, the study by Spatz et al. had a small sample size, and neither Spatz et al. nor Rosenstock et al. factored in some important variables such as blood pressure, LVEF, body mass index (BMI), and albumin and uric acid levels, which may have led to different results.

The pathophysiologic mechanisms of CIAKI have not been fully clarified, although several factors, including oxidative stress, endothelial dysfunction, and free radical damage, have been pointed out [29, 30]. Substantial research links these processes to elevations in plasma glucose [31]. Experimental and clinical evidence has shown that acutely increased plasma glucose levels suppress flow-mediated vasodilatation and promote vascular damage through increased production of oxygen-derived free radicals [32, 33]. Moreover, acute hyperglycemia may induce osmotic diuresis, resulting in volume depletion and dehydration and further increasing AKI risk and severity [34]. Therefore, conditions associated with hyperglycemia, like diabetes, may exacerbate through oxidative stress the deleterious effects of contrast agents on kidney function and thus increase CIAKI risk. In this regard, evidence indicates that production of reactive oxygen species (ROS) after contrast media exposure reduces renal nitric oxide (NO) levels and potentiates vasoconstriction mediated by adenosine, angiotensin II (AngII), and endothelin I, among others, thus favoring renal ischemia and hypoxia [35]. Theoretically, these effects can be counteracted by ACEIs, which increase the synthesis and bioactivity of NO and other vasodilator factors [36, 37]. Although such a protective role of RAAS inhibitors against CIAKI appears to be supported by the aforementioned study results from Gupta et al. [25], our results as well as others’ suggest that RAAS blockade actually increases CIAKI risk in high-risk diabetic patients. Other purported benefits of ACEIs on renal function include relieving AngII-mediated vasoconstriction of efferent glomerular arterioles, thus decreasing both glomerular venous pressure and glomerular filtration rate [38]. However, it was shown that due to these effects, the clearance of contrast media becomes slower, which may lead to persistent kidney damage [39]. AngII induces the production of transforming growth factor-β1 (TGF-β1), an important mediator of renal fibrosis that showed however protective effects against renal proximal tubule cell necrosis [40, 41]. Thus, it was speculated that by inhibiting AngII production, ACEIs would negate the potentially protective effect of TGF-β1 in the kidney and favor CIAKI onset [42].

We found that the incidence of CIAKI peaked during the initial 48 h post-CAG/PCI, but new cases were still detected up to 72 h following the procedure. Data from Reddan et al. [43] and Davidson et al. [44] showed that a single 24-h measurement would have missed 58.2% of CIAKI cases that were otherwise detected over a 48-h time frame. Based on these considerations, we chose a 72-h window to evaluate the incidence of CIAKI in our study. We also found that CIAKI incidence was higher in elderly patients. This is not surprising, since with increased age there are structural changes in the kidney (i.e. cortical thinning, volume decrease, reduction in the number of functional nephrons and glomeruli, arteriosclerosis, etc.) that contribute to reduce GFR [45]. As in previous studies, factors such as CHF, anemia, CKD, hypoalbuminemia, multi-vessel disease, and myocardial infarction were also correlated with higher CIAKI incidence [46–48]. Hyperuricemia was also an independent risk factor for CIAKI in our study. A recent report suggested that a serum uric acid value of 5.55 mg/dl or more was the best predictor of CIAKI [48]. Hyperuricemia was found to contribute to renal dysfunction by reducing NO availability, activating the local RAAS in the kidney, and promoting ROS production and oxidative stress [49]. Additionally, we found that proteinuria, which has been largely overlooked as a risk factor until recently, was also associated with CIAKI incidence. Evidence indicates that proteinuric patients are less tolerant to renal hemodynamic changes and more susceptible to injury caused by nephrotoxic substances such as iodinated contrast agents [50]. Therefore, hyperuricemia and proteinuria should be considered for risk assessment in PCI patients. The ESUR Contrast Media Safety Committee has pointed out that patients who need contrast enhancement tests should be asked if they have a history of kidney disease, kidney surgery, proteinuria, diabetes, hypertension, and gout [51]. Meanwhile, several prevention strategies (i.e. lower contrast agent volumes, pre-procedural hydration, and N-acetylcysteine and statin supplementation), are commonly adopted in clinical practice [52].

In summary, our data indicates that the incidence of CIAKI in diabetic patients might be increased after RAAS blockade, especially in high-risk subgroups such as the elderly, higher volume of contrast agent, and eGFR <60 mL/min/1.73 m^2^. We also found that worsening heart failure occurred more frequently in patients who developed CIAKI than in those who did not (2.3% vs. 1.0%, respectively) although this trend did not attain significance. Of note, patients in the ACEIs/ARBs group had fewer adverse cardiovascular events than control patients (1.8% vs. 4.1%, *P*=0.016), which is consistent with the cardioprotective effects of RAAS inhibitors [9].

Our study has some limitations. It was retrospective in nature, and although PSM has been used to reduce potential confounding and selection biases, there are still some important factors that were not addressed. At the same time, a large number of unmatched patients were eliminated during PSM, so we were unable to draw conclusions regarding this patient set. Also, the long-term prognosis of CIAKI in our patient cohorts is still not known, therefore follow-up data on renal and cardiovascular outcomes need to be collected. Third, our analysis did not discriminate outcomes based on ACEIs/ARBs dosage and types, which may have provided a more precise account of the impact of RAAS blockade on CIAKI incidence. Nevertheless, this is the first multi-center research study addressing perioperative use of RAAS blockers in high-risk patients with diabetes exposed to contrast media during CAG/PCI procedures. In the future, prospective randomized clinical trials should provide a clearer picture of the effects of RAAS inhibition therapy on CIAKI and other early and long-term outcomes.

## MATERIALS AND METHODS

### Study population

This multi-center, retrospective study included patients from Nanjing First Hospital, Sir Run Run Hospital at Nanjing Medical University, Xu Zhou Medical University Hospital, and Affiliated Shu Yang Hospital. From January 2014 to July 2017, patients with diabetes that underwent CAG and PCI in one of the above four medical centers were screened for inclusion in the study. Patients were excluded based on: (1) allergy to contrast agents; (2) missing periprocedural creatinine data; (3) ESRD requiring dialysis before surgery; (4) use of metformin, nonsteroidal anti-inflammatory drugs, aminoglycoside antibiotics or other nephrotoxic drugs within 48 h before surgery; (5) repeated exposure to contrast medium within the last 2 weeks; (6) history of cardiogenic shock; (7) bilateral renal artery stenosis or hyperkalemia; (8) acute diabetic complications including diabetic ketoacidosis, non-ketonic hyperosmolar coma, and diabetic lactic acidosis; and (9) acute kidney injury before surgery. The study was performed in accordance with the Declaration of Helsinki on human research. Our hospitals approved this study and waived the requirement for informed consent because of its retrospective design. On admission, all patients were administered aspirin, clopidogrel, and statin. Intravenous hydration with 500 ml of 0.9% sodium chloride was administered at least 6 h before and after exposure to the contrast agent.

### ACEIs/ARBs administration

Prescription drug history was assessed by reviewing the patient medication record systems. Patients who received RAAS blockers 24 h prior to and over 3 days following PCI treatment were included in the ACEIs/ARBs group, while patients who did not receive RAAS blockers in the perioperative period were included in the control group.

### Data collection

Demographic and laboratory data, including age, gender, BMI, blood pressure before surgery, preoperative complications, coronary artery disease, contrast agent administration, and periprocedural biochemical indicators were obtained from medical records. Perioperative medication (ACEIs/ARBs, β-blockers, calcium channel blockers, diuretics, insulins, and oral hypoglycemic agents) were retrieved from patients’ medication records at each institution. Baseline creatinine level was defined as the lowest creatinine value within 7 days before surgery and we collected all recorded creatinine values 72 h postoperatively.

### CAG and PCI

Coronary artery disease was defined as presence of at least one coronary stenosis >50%. CAG was performed via transradial or transfemoral routes using the Seldinger technique, by placing a 6-F catheter into the radial or femoral artery followed by conventional positioning of a contrast catheter in the coronary ostia. PCI was performed by balloon dilatation or stenting; stent type was chosen at the discretion of the surgeon.

### Clinical endpoints

The primary endpoint was CIAKI, defined as a relative increase in serum creatinine ≥25% or an absolute increase ≥ 44 μmol/l (0.5 mg/dl) from baseline within 72 h after contrast agent exposure, excluding other factors that could cause acute kidney injury [53, 54]. Other defining criteria for CIAKI were an absolute increase in serum creatinine ≥0.3 mg/dl (26.4 μmol/l), or a relative increase ≥50% over baseline within 48 h [55]. Additional endpoints were dialysis, adverse cardiovascular events such as myocardial infarction (including new-onset or recurrence), worsening heart failure, stroke, and death, and length of in-hospital stay.

### Other definitions

Based on the New York Heart Disease Association (NYHA) classification system, diagnosis of CHF was established for patients with NYHA class III or higher. A cut-off age of 70 years was chosen to classify the elder. Anemia was defined as hemoglobin <110 g/l in females or <120 g/l in males. Hypoalbuminemia was defined as albumin <35 g/l. Hyperuricemia was defined as uric acid >420 μmol/l. Hypercholesterolemia was defined as cholesterol >5.17 mmol/l. Hypertriglyceridemia was defined as triglycerides >2.3 mmol/l. According to the American Diabetes Association Practice Guidelines, diabetes mellitus (DM) was defined by fasting blood glucose concentration ≥126 mg/dl, or a clinical diagnosis of DM with dietary, oral, or insulin treatment [56]. Hypertension was defined as office SBP values >_140 mmHg and/or diastolic BP (DBP) values >_90 mmHg [57]. CKD was defined as estimated GFR (eGFR) <60 ml/min/1.73 m^2^, proteinuria (defined as trace or greater by dipstick), or both on at least 2 occasions ≥3 months apart [58, 59].

### Data analysis

We used propensity score matching (PSM), which allows reducing potential confounding and selection biases, and the conditional logistic model to examine the effect of ACEIs/ARBs on the incidence of CIAKI. First, we analyzed the data from a single center (Nanjing First Hospital) to obtain preliminary results. Then, we validated those results using data from the other three hospitals. Finally, we combined all the data and the matched data from each center separately to arrive at the final results (Figure 1).

Prior to PSM analysis, differences between ACEIs/ARBs and control groups were compared using chi-square test or Fisher’s exact test for categorical variables or unpaired *t* test for continuous variables, as appropriate. Continuous variables were expressed as the mean ± SD, and categorical variables were expressed as percentages. Multivariate logistic regression analysis with the backward stepwise method was employed to determine independent risk factors for CIAKI. Adjusted variables were female gender, age >70 years, BMI, diabetes history, hypertension, CHF, prior myocardial infarction, AMI, angina pectoris, single- or multi-vessel coronary artery disease, types and volumes of contrast agents used, preoperative systolic and diastolic blood pressure, β-blockers, diuretics, CCB, insulins, hypoglycemic agents, preoperative glucose, baseline creatinine, urea nitrogen, eGFR, CKD, proteinuria, anemia, albumin <35 g/L, uric acid >420 μmol/l, hypercholesterolemia, hypertriglyceridemia, high-density lipoprotein (HDL), low-density lipoprotein (LDL), and LVEF <40%.

To analyze the correlation between ACEs/ARBs use and CIAKI incidence, we first applied a multivariable logistic regression analysis model to predict the probability of receiving ACEs/ARBs treatment. Then, the propensity score for each patient was calculated by using the covariates listed in Table 1. Finally, the nearest neighbor matching algorithm was used to match ACEs/ARBs users with non-users in a 1:1 ratio (unmatched patients were excluded from analysis). After PSM was finished, the balance of covariates between the two groups was assessed through paired *t* tests and McNemar’s tests as appropriate for continuous and categorical variables. Once the matched data was adjusted for the effect of the covariates on the outcome, the relationship between RAAS blockade and CIAKI was determined by conditional logistic regression. McNemar’s tests were used to contrast the main effect on CIAKI between the ACEIs/ARBs and control groups. Nonparametric tests were used to compare the incidence of CIAKI in both patient groups within 24, 48, and 72 h post-CAG/PCI. The effect of RAAS blockade on CIAKI was also evaluated in various subgroups (defined by gender, age, diabetes history, contrast volume, eGFR, LVEF, anemia, albumin, uric acid, or proteinuria) using matched data and adjusted for the propensity scores.

Data are expressed as odds ratio (OR) with 95% confidence interval (CI) and percentage, and *P*<0.05 was considered significant. SPSS version 23.0 (SPSS, Chicago, IL, USA) and R software (version 3.2.1; http://www.r-project.org/) were used for statistical analyses.

## Supplementary Material

Supplementary Figure 1

Supplementary Tables
