# Supplementary material for: Renin-angiotensin-aldosterone system blockade is associated with higher risk of contrast-induced acute kidney injury in patients with diabetes
Source: Aging (Albany NY). 2020 Apr 2;12(7):5858–77. doi: 10.18632/aging.102982 (PMC7185147; doi:10.18632/aging.102982)
Supplement: Supplementary Figure 1 [file aging-12-102982-s001..pdf]

SUPPLEMENTARY FIGURE

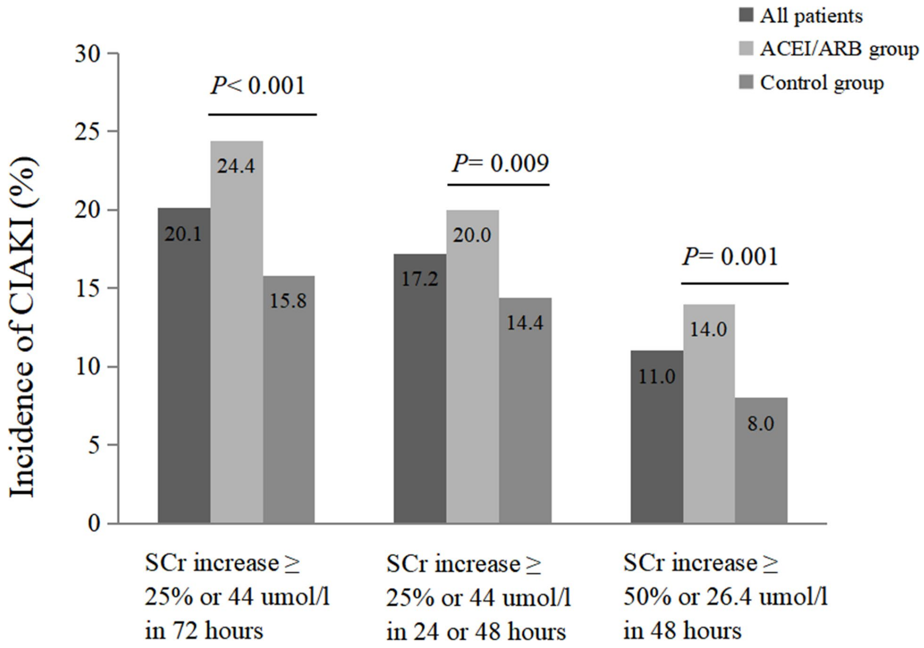

Supplementary Figure 1. Impact of RAAS inhibition on CIAKI incidence. Incidence of CIAKI in the PSM-matched cohort under different definitions (after merging matched data from each center, 659 pairs of patients).
