# Supplementary material for: Renin-angiotensin-aldosterone system blockade is associated with higher risk of contrast-induced acute kidney injury in patients with diabetes
Source: Aging (Albany NY). 2020 Apr 2;12(7):5858–77. doi: 10.18632/aging.102982 (PMC7185147; doi:10.18632/aging.102982)
Supplement: Supplementary Tables [file aging-12-102982-s002..pdf]

SUPPLEMENTARY TABLES

Supplementary Table 1. Baseline characteristics of patients in the first center before and after propensity score matching.

Variable	Before matching			After propensity matching		
	ACEI/ARB group (n=913)	Control group (n=612)	*P* value	ACEI/ARB group (n=461)	Control group (n=461)	*P* value
**Demographics:**						
Female	299(32.7)	197(32.2)	0.819	144(31.2)	147(31.9)	0.884
Age (yrs)	67±10	66±11	0.557	67±10	67±10	0.638
BMI (kg/m^2)	25.5±3.1	24.8±3.0	0.310	25.0±3.0	25.1±3.0	0.816
**Medical history:**						
Diabetes history (yrs)	8.5±5.6	8.2±5.6	0.972	8.4±5.3	8.5±5.7	0.897
Hypertension	829(90.8)	376(61.4)	<0. 001	378(82.0)	367(79.6)	0.090
CHF	148(16.2)	87(14.2)	0.290	76(16.5)	70(15.2)	0.656
CKD	181(13.8)	108(11.6)	0.125	68(14.8)	20(15.2)	0.923
AMI	184(20.2)	142(23.2)	0.155	92(20.0)	102(22.1)	0.456
Prior myocardial infarction	81(8.9)	44(7.2)	0.241	35(7.6)	37(8.0)	0.904
Stable angina pectoris	62(6.8)	54(8.8)	0.142	40(8.7)	37(8.0)	0.807
Unstable angina	311(34.1)	197(32.2)	0.447	161(34.9)	152(33.0)	0.582
**CAG and PCI:**						
Multi-vessel disease	522(57.2)	344(56.2)	0.709	265(57.5)	257(55.7)	0.647
Single-vessel disease	307(33.6)	198(32.4)	0.605	155(33.6)	155(33.6)	1.000
Preoperative SBP (mmHg)	137±17	130±16	0.021	133±15	133±16	0.933
Preoperative DBP (mmHg)	80±12	77±11	0.758	78±10	78±11	0.362
**Contrast agent:**						
Nonionic iso-osmolar	504(55.2)	343(56.0)	0.745	269(58.4)	264(57.3)	0.781
Nonionic low-osmolar	391(43.2)	262(42.8)	0.894	188(40.8)	191(41.4)	0.889
Volume of contrast agent (mL)	194±73	189±70	0.683	190±72	192±71	0.672
**Medications :**						
Β-blocker	550(60.2)	318(52.0)	0. 001	256(55.5)	247(53.6)	0.589
Diuretics	144(15.8)	100(16.3)	0.767	69(15.0)	75(16.3)	0.643
CCB	227(24.9)	129(21.1)	0.087	121(26.2)	122(26.5)	1.000
Insulins	454(49.7)	315(51.5)	0.504	232(50.3)	231(50.1)	1.000
Oral hypoglycemic agent	491(53.8)	326(53.3)	0.845	255(55.3)	254(55.1)	1.000
**Pre-procedural laboratory determinations:**						
Glucose (mmol/L)	9.8±3.7	9.7±3.7	0.879	9.8±3.6	9.6±3.6	0.489
Baseline creatinine (umol/L)	79.3±29.6	79.0±36.3	0.309	79.3±31.6	80.2±32.0	0.678
eGFR (mL/min/1.73 m^2)	82.9±20.7	84.5±21.2	0.657	83.2±20.8	82.5±20.8	0.581
Proteinuria	147(16.1)	60(9.8)	<0. 001	39(8.5)	46(10.0)	0.489
Hemoglobin (g/L)	131.9±16.7	131.8±16.7	0.961	131±17	132±17	0.997
Albumin (g/L)	38.7±3.7	38.4±4.0	0.284	38.5±3.7	38.4±4.0	0.510
Uric acid (umol/L)	344.1±113.4	335.8±111.5	0.438	343.9±115.7	342.5±109.0	0.847
Total cholesterol (mmol/L)	3.9±1.1	3.9±1.8	0.178	3.9±1.1	3.8±1.2	0.608
Triglycerides (mmol/L)	1.8±1.4	1.7±1.5	0.659	1.8±1.4	1.8±1.6	0.818
HDL (mmol/L)	0.99±0.24	0.99±0.24	0.688	0.98±0.23	0.99±0.23	0.724
LDL (mmol/L)	2.30±0.92	2.32±0.95	0.445	2.30±0.91	2.27±0.90	0.586
LVEF (%)	59.1±10.0	58.7±10.0	0.481	59.1±10.1	58.9±9.8	0.822

Abbreviations: ACEI, angiotensin-converting enzyme inhibitor; ARB, angiotensin receptor blocker; BMI, body mass index; CKD, chronic kidney disease; CHF, congestive heart failure; AMI, acute myocardial infarction; CCB, calcium channel blocker; eGFR, estimated glomerular filtration rate; HDL, high-density lipoprotein; LDL, low-density lipoprotein; LVEF, left ventricular ejection fraction.

Supplementary Table 2. Baseline characteristics of patients in other three centers before and after propensity score matching.

Variable	Before matching			After propensity matching		
	ACEI/ARB group (n=397)	Control group (n=318)	*P* value	ACEI/ARB group (n=198)	Control group (n=198)	*P* value
**Demographics:**						
Female	114(35.8)	159(40.1)	0.251	66(33.3)	68(34.3)	0.921
Age (yrs)	65±10	64±11	0.256	66±10	65±11	0.442
BMI (kg/m^2)	25.3±2.8	25.2±3.0	0.898	25.3±2.7	25.4±2.8	0.777
**Medical history:**						
Diabetes history (yrs)	7.7±6.3	8.3±6.9	0.272	8.5±6.3	8.8±7.4	0.653
Hypertension	317(79.8)	171(53.8)	**<0. 001**	146(73.7)	136(68.7)	0.289
CHF	47(11.8)	45(14.2)	0.359	29(14.6)	24(12.1)	0.551
CKD	44(11.1)	26(8.2)	0.194	23(11.6)	21(10.6)	0.878
AMI	90(22.7)	80(25.2)	0.438	45(22.7)	38(19.2)	0.483
Prior myocardial infarction	25(6.3)	20(6.3)	0.997	15(7.6)	15(7.6)	1.000
Stable angina pectoris	19(4.8)	12(3.8)	0.509	8(4.0)	11(5.6)	0.648
Unstable angina	214(53.9)	126(39.6)	**<0. 001**	88(44.4)	90(45.5)	0.920
**CAG and PCI:**						
Multi-vessel disease	275(69.3)	168(52.8)	**<0. 001**	113(57.1)	113(57.1)	1.000
Single-vessel disease	83(20.9)	95(29.9)	**0.006**	55(27.8)	55(27.8)	1.000
Preoperative SBP (mmHg)	137±18	132±18	0.333	136±18	136±18	0.903
Preoperative DBP (mmHg)	81±12	78±11	0.705	80±11	80±11	0.755
**Contrast agent:**						
Nonionic iso-osmolar	134(33.8)	101(31.8)	0.573	79(39.9)	67(33.8)	0.251
Nonionic low-osmolar	263(66.2)	217(68.2)	0.573	119(60.1)	131(66.2)	0.251
Volume of contrast agent (mL)	161±77	159±76	0.941	166±87	165±84	0.889
**Medications :**						
B-blocker	293(73.8)	121(38.1)	**<0. 001**	109(55.1)	100(50.5)	0.321
Diuretics	186(46.9)	43(13.5)	**<0. 001**	39(19.7)	39(19.7)	1.000
CCB	99(24.9)	84(26.4)	0.653	56(28.3)	57(28.8)	1.000
Insulins	130(32.7)	104(32.7)	0.991	75(37.9)	69(34.8)	0.621
Oral hypoglycemic agent	273(68.8)	170(53.5)	**<0. 001**	114(57.6)	114(57.6)	1.000
**Pre-procedural laboratory determinations:**						
Glucose (mmol/L)	9.1±3.3	9.4±3.9	**0.033**	9.3±3.6	9.2±3.7	0.770
Baseline creatinine (umol/L)	72.8±27.6	71.6±29.2	0.451	75.8±29.7	74.6±33.9	0.703
eGFR (mL/min/1.73 m^2)	88.0±20.4	89.8±19.8	0.456	86.3±20.8	87.8±20.8	0.475
Proteinuria	60(15.1)	45(14.2)	0.718	23(11.6)	24(12.1)	1.000
Hemoglobin (g/L)	132.4±16.8	134.2±16.9	0.648	133±16	136±17	0.117
Albumin (g/L)	40.6±4.5	40.0±4.9	0.401	40.1±4.2	40.4±5.2	0.467
Uric acid (umol/L)	326.4±102.9	313.3±105.5	0.447	329.1±103.5	320.4±101.6	0.388
Total cholesterol (mmol/L)	4.2±1.2	4.1±1.1	0.056	4.0±1.22	4.1±1.1	0.548
Triglycerides (mmol/L)	1.9±1.5	1.8±1.4	0.318	2.0±1.7	1.9±1.2	0.211
HDL (mmol/L)	1.07±0.29	1.06±0.29	0.695	1.02±0.25	1.04±0.27	0.625
LDL (mmol/L)	2.41±0.93	2.37±0.92	0.596	2.35±0.92	2.37±0.95	0.800
LVEF (%)	57.1±9.4	58.4±9.0	0.160	57.9±9.8	58.4±9.0	0.652

Abbreviations: ACEI, angiotensin-converting enzyme inhibitor; ARB, angiotensin receptor blocker; BMI, body mass index; CKD, chronic kidney disease; CHF, congestive heart failure; AMI, acute myocardial infarction; CCB, calcium channel blocker; eGFR, estimated glomerular filtration rate; HDL, high-density lipoprotein; LDL, low-density lipoprotein; LVEF, left ventricular ejection fraction.

Supplementary Table 3. Baseline characteristics of patients before and after merging each center matched.

Variable	Before matching			After merging each center matched			The rest after matching		
	ACEI/ARB group (n=1310)	Control group (n=930)	*P* value	ACEI/ARB group (n=659)	Control group (n=659)	*P* value	ACEI/ARB group (n=651)	Control group (n=271)	*P* value
**Demographics:**									
Female	458(35.0)	311(33.4)	0.455	210(31.9)	215(32.6)	0.815	248(38.1)	96(35.4)	0.445
Age (yrs)	66±10	66±11	0.238	66±10	67±10	0.982	65±10	63±11	0.356
BMI (kg/m^2)	25.4±3.1	24.9±3.0	0.381	25.1±2.9	25.2±2.9	0.726	25.7±3.2	24.4±3.0	0.239
**Medical history:**									
Diabetes history (yrs)	8.2±5.8	8.3±6.0	0.433	8.4±5.6	8.6±6.2	0.704	8.0±6.1	7.5±5.6	0.287
Hypertension	1146(87.5)	547(58.8)	<0. 001	524(79.5)	503(76.3)	0.053	622(95.5)	44(16.2)	<0. 001
CHF	195(14.9)	132(14.2)	0.648	105(15.9)	94(14.3)	0.445	90(13.8)	38(14.0)	0.937
CKD	181(13.8)	108(11.6)	0.125	91(13.8)	91(13.8)	1.000	90(13.8)	17(6.3)	0.001
AMI	274(20.9)	222(23.9)	0.097	137(20.8)	140(21.2)	0.893	137(21.0)	82(30.3)	0.003
Prior myocardial infarction	106(8.1)	64(6.9)	0.287	50(7.6)	52(7.9)	0.919	56(8.6)	12(4.4)	0.027
Stable angina pectoris	81(6.2)	66(7.1)	0.390	48(7.3)	48(7.3)	1.000	33(5.1)	18(6.6)	0.342
Unstable angina	525(40.1)	323(34.7)	0.010	249(37.8)	242(36.7)	0.734	276(42.4)	81(29.9)	<0. 001
**CAG and PCI:**									
Multi-vessel disease	797(60.8)	512(55.1)	0.006	378(57.4)	370(56.1)	0.703	419(64.4)	142(52.4)	0. 001
Single-vessel disease	390(29.8)	293(31.5)	0.380	210(31.9)	210(31.9)	1.000	180(27.6)	83(30.6)	0.362
Preoperative SBP (mmHg)	137±17	131±17	0.017	134±16	133±17	0.887	141±18	124±16	0.005
Preoperative DBP (mmHg)	80±12	78±11	0.685	78±11	79±11	0.355	83±13	75±10	0.004
Contrast agent:									
Nonionic iso-osmolar	638(48.7)	444(47.7)	0.654	348(52.8)	331(50.2)	0.355	290(44.5)	113(41.7)	0.427
Nonionic low-osmolar	657(50.2)	479(51.5)	0.528	307(46.6)	322(48.9)	0.418	350(53.8)	157(57.9)	0.247
Volume of contrast agent (mL)	184±76	179±74	0.681	183±77	184±76	0.800	185±74	166±67	0.828
**Medications :**									
B-blocker	843(64.4)	439(47.2)	<0. 001	365(55.4)	347(52.7)	0.313	478(73.4)	92(33.9)	<0. 001
Diuretics	330(25.2)	143(15.4)	<0. 001	108(16.4)	114(17.3)	0.701	222(34.1)	29(10.7)	<0. 001
CCB	326(24.9)	213(22.9)	0.280	177(26.9)	179(27.2)	0.950	149(22.9)	34(12.5)	<0. 001
Insulins	584(44.6)	419(45.1)	0.824	307(46.6)	300(45.5)	0.736	277(42.5)	119(43.9)	0.704
Oral hypoglycemic agent	764(58.3)	496(53.3)	0.019	369(56.0)	368(55.8)	1.000	395(60.7)	128(47.2)	<0. 001
**Pre-procedural laboratory determinations:**									
Glucose (mmol/L)	9.6±3.6	9.6±3.9	0.183	9.6±3.6	9.5±3.6	0.460	9.5±3.5	9.9±4.1	0.038
Baseline creatinine (umol/L)	77.3±29.2	76.5±34.2	0.750	78.2±31.1	78.5±32.7	0.895	76.3±27.0	71.7±37.4	0.332
eGFR (mL/min/1.73 m^2)	84.4±20.8	86.3±20.9	0.419	84.2±20.8	84.1±20.9	0.945	84.7±20.7	91.8±19.6	0.002
Proteinuria	207(15.8)	105(11.3)	0.002	62(9.4)	70(10.6)	0.519	145(22.3)	35(12.9)	0. 001
Hemoglobin (g/L)	132.1±16.7	132.6±16.8	0.831	132±17	133±17	0.382	132±17	132±16	0.568
Albumin (g/L)	39.3±4.0	38.9±4.4	0.260	39.0±3.9	39.0±4.5	0.954	39.6±4.1	38.7±4.0	0.834
Uric acid (umol/L)	338.7±110.6	328.1±109.9	0.273	339.4±112.3	335.9±107.2	0.539	338.0±109.0	309.4±114.3	0.277
Total cholesterol (mmol/L)	4.0±1.2	4.0±1.2	0.899	3.9±1.2	3.9±1.1	0.924	4.0±1.2	4.1±1.2	0.077

Triglycerides (mmol/L)	1.9±1.5	1.8±1.4	0.318	1.8±1.4	1.8±1.5	0.766	1.9±1.6	1.7±1.1	0.054
HDL (mmol/L)	1.01±0.26	1.02±0.26	0.783	1.00±0.24	1.00±0.25	0.567	1.03±0.28	1.05±0.29	0.626
LDL (mmol/L)	2.33±0.92	2.34±0.94	0.756	2.31±0.92	2.30±0.91	0.751	2.35±0.93	2.44±0.98	0.674
LVEF (%)	58.4±9.8	58.6±9.7	0.495	59.7±10.0	58.7±9.6	0.957	58.2±9.6	58.2±9.9	0.532

Abbreviations: ACEI, angiotensin-converting enzyme inhibitor; ARB, angiotensin receptor blocker; BMI, body mass index; CKD, chronic kidney disease; CHF, congestive heart failure; AMI, acute myocardial infarction; CCB, calcium channel blocker; eGFR, estimated glomerular filtration rate; HDL, high-density lipoprotein; LDL, low-density lipoprotein; LVEF, left ventricular ejection fraction.

Supplementary Table 4. The relationship between ACEI/ARB and CIAKI before and after matching (using the data before and after merging each center matched).

Definitions	Unmatched cohort		Matched cohort	
	OR (95% CI)*	*P* value*	OR (95% CI)**	*P* value**
Primary CIAKI end point:				
SCr increase ≥ 25% or 44 umol/l in 72 hours	1.757 (1.401-2.203)	<0.001	1.706 (1.295-2.246)	<0.001
Other defining criteria for CIAKI:				
SCr increase ≥ 25% or 44 umol/l in 24 or 48 hours	1.583 (1.259-1.990)	<0.001	1.484 (1.111-1.982)	0.008
SCr increase ≥ 50% or 26.4 umol/l in 48 hours	2.009 (1.510-2.673)	<0.001	1.814 (1.268-2.594)	0.001

* Multivariable analysis was applied in the unmatched cohort. OR and 95% confidence interval (CI) were obtained by adjusting variables.

** Conditional logistic model was applied in the matched cohort, OR with 95% confidence interval (CI) was obtained.

Abbreviations: SCr, serum creatinine; CIAKI, contrast-induced acute kidney injury.

Supplementary Table 5. Comparison of in-hospital outcomes between the control group and the ACEI/ARB group in the matched cohort (using the data after merging each center matched, 659 pairs of patients).

Outcome	Control group (n=659)	ACEI/ARB group (n=659)	*P* value
CIAKI, n (%)	104 (15.8)	161 (24.4)	< 0.001
Dialysis, n (%)	0	1 (0.2)	-
Deaths, n (%)	2(0.3)	0	-
Worsening heart failure, n (%)	5 (0.8)	3 (0.5)	0.727
Myocardial infarction, n (%)	7 (1.1)	1 (0.2)	0.070
Stroke, n (%)	0	1(0.2)	-
Overall adverse cardiovascular events (at least 1)	14(2.1)	5(0.8)	0.039
Length of in-hospital stay, d	7.93±4.13	8.34±4.20	0.071

Abbreviation: CIAKI, contrast-induced acute kidney injury
